# Assessment of China’s contributions to the Regional Network for Asian Schistosomiasis and Other Helminth Zoonoses: a questionnaire survey

**DOI:** 10.1186/s41256-021-00186-3

**Published:** 2021-02-17

**Authors:** Hong-Mei Li, Ying-Jun Qian, Kun Yang, Wei Ding, Lu-Lu Huang, Xue-Jiao Ma, Lei Duan, Duo-Quan Wang, Ya-Yi Guan, Ning Xiao, Xiao-Nong Zhou

**Affiliations:** 1 National Institute of Parasitic Diseases, Chinese Center for Disease Control and Prevention; Chinese Center for Tropical Diseases Research; WHO Collaborating Centre for Tropical Diseases; National Center for International Research on Tropical Diseases, Ministry of Science and Technology; Key Laboratory of Parasite and Vector Biology, Ministry of Health, Shanghai, 200025 China; 2 Jiangsu Institute of Parasitic Diseases, Wuxi, 214064 China

**Keywords:** China, RNAS^+^, Assessment, Schistosomiasis, Regional cooperation, Contribution

## Abstract

**Background:**

The Regional Network for Asian Schistosomiasis and Other Helminth Zoonoses (RNAS^+^) was established in 1998, which has developed close partnerships with Asian countries endemic for schistosomiasis and other helminthiasis in Asia. RNAS^+^ has provided an ideal regional platform for policy-makers, practitioners and researchers on the prevention, control and research of parasitic diseases in Asian countries. China, one of the initiating countries, has provided significant technical and financial support to the regional network. However, its roles and contributions have not been explored so far. The purpose of this study was to assess China's contributions on the supporting of RNAS^+^ development.

**Methods:**

An assessment research framework was developed to evaluate China’s contributions to RNAS^+^ in four aspects, including capacity building, funding support, coordination, and cooperation. An anonymous web-based questionnaire was designed to acquire respondents’ basic information, and information on China’s contributions, challenges and recommendations for RNAS^+^development. Each participant scored from 0 to 10 to assess China’s contribution: “0” represents no contribution, and “10” represents 100% contribution. Participants who included their e-mail address in the 2017–2019 RNAS^+^ annual workshops were invited to participate in the assessment.

**Results:**

Of 71 participants enrolled, 41 responded to the survey. 37 (37/41, 90.24%) of them were from RNAS^+^ member countries, while the other 4 (4/41, 9.76%) were international observers. Most of the respondents (38/41, 92.68%) were familiar with RNAS^+^. Respondents reported that China’s contributions mainly focused on improving capacity building, providing funding support, coordination responsibility, and joint application of cooperation programs on RNAS^+^ development. The average scores of China’s contributions in the above four fields were 8.92, 8.64, 8.75, and 8.67, respectively, with an overall assessment score of 8.81 (10 for a maximum score). The challenge of RNAS^+^ included the lack of sustainable funding, skills, etc. and most participants expressed their continual need of China’s support.

**Conclusions:**

This survey showed that China has played an important role in the development of RNAS^+^ since its establishment. This network-type organization for disease control and research can yet be regarded as a great potential pattern for China to enhance regional cooperation. These findings can be used to promote future cooperation between China and other RNAS^+^ member countries.

## Background

Schistosomiasis, a serious infectious disease caused by blood flukes, occurs in a total of 78 endemic countries worldwide [1]. Until now, except in Japan where the disease has been eliminated, other Asian endemic countries are still at risk of transmission, including China, the Philippines, Indonesia, Laos, Cambodia, Malaysia, Myanmar, and so on [2, 3]. To achieve the goal set by the United Nations in the 2030 Agenda for Sustainable Development Goals (SDGs) of eliminating schistosomiasis by 2030, a comprehensive, multi-sectoral, and multifaceted approach across countries is needed to control, and eventually eliminate, the disease in Asia [4, 5].

Efforts were made to initiate a collaboration between national research institutions and research groups within and outside Asian countries 20 years ago [6, 7]. The Regional Network on Asian Schistosomiasis (RNAS) was originated during a discussion session at an international workshop held in China in 1996, and was planned to build a formal network during another international seminar in China in 1998. RNAS was formally established during the first workshop of the RNAS network in the Philippines in 2000 [2], which aimed at uniting intersectoral, interregional, and international collaborations for multidisciplinary organizations (human medicine and veterinary medicine) and experts (researchers, practitioners, and policy-makers) in various fields from Asian disease endemic countries. The initiating areas of work included collaborative research, surveillance, and control of schistosomiasis in Asia, and the network also advanced the technology for the control of *Schistosoma japonicum* [8, 9]. In the fifth RNAS workshop in 2005, its focus was expanded from schistosomiasis to include other diseases and conditions, including cysticercosis, clonorchiasis, opisthorchiasis, paragonimiasis and fascioliasis, caused by helminthiasis treatable with praziquantel, thus forming the Regional Network for Asian Schistosomiasis and Other Helminth Zoonoses (RNAS^+^) of what is today [2, 10, 11].

The developmental pathway of RNAS^+^ started with only two countries (China and the Philippines) in 2000 [2, 4]. Since then, its members have gradually increased to 11 countries (China, the Philippines, Cambodia, Indonesia, Laos, Thailand, South Korea, Japan, Vietnam, Myanmar, and Malaysia) in 2018 [2, 4]. Up to date, over 90% of research institutions and disease control organizations working on schistosomiasis in the 11-member countries have participated in RNAS^+^ activities [2, 12].

After two-decade operation, the RNAS^+^ has mainly been devoted to the following four areas. First, an operational mode was developed for operating the RNAS^+^ [11, 13]. An executive committee was formed to manage the RNAS^+^, which is composed of a chairman, a vice-chairman, several executive members, and several international observers. A rotating chairman system was adopted to elect the chairman. Second, a mechanism for information exchange and dissemination was developed by annually hosting academic workshops and training courses [12]. To date, 20 workshops have been held in different member countries, which have contributed to improved neglected tropical diseases (NTDs) control programs in the member countries [2]. Third, some multi-country cooperative research projects have been implemented, through which scientists from different countries have learned from one another to improve their relevant technologies for the application of serodiagnostics, ultrasound examination, mollusciciding, and surveillance response systems [14, 15]. Fourth, capacity building has been improved by more than a dozen of training courses in the field of diagnosis, molecular biology, geographic information systems, and ethical issues, specifically desingned for the young generations of the  member countries [4, 16].

Some studies assessed the performance and improvement on health system, capacity building, quality management, health technology [17–20], and so on. However, no published work can be found on the assessment of the contributions of any member country in regional networks on disease prevention and control. After a literature review, a qualitative study was conducted by Furnival et al.(2018) to assess the capacity improvement of six UK healthcare organizations, using semi-structured interviews, policy documents, and assessment reports as their data sources [17]. A self-assessment questionnaire proposed by the World Health Organization (WHO) was used to assess the quality of the Estonian health system and comprised four domains (policy, organization, methods, and resources) [18]. A scoring criteria were developed to assess the quality of program theory [19]. A systematic review was used to evaluate the screening performance of maternal serum and ultrasound markers in detecting Down syndrome [20]. Moreover,  methods for assessing the contributions of one country to a network to which it belongs are scant. Some researchers have recently explored a method to assess in-kind contributions in a donor-funded health capacity-building program in Africa, which estimates the monetary value of those contributions [21].

 RNAS^+^ celebrated its 20th anniversary in 2018 in Shanghai, China [2]. Although China along with other member countries have provided great technical and resource support on the RNAS^+^ development, China’s roles and contributions have not been explored systematically. This study aimed to perform an assessment of China’s roles and contributions in the developmental pathway of RNAS^+^.

## Methods

### Research design

In this study, an assessment research framework was planned to assess China’s contributions according to the four major achievements of RNAS^+^in the past 20 years. This framework covers the following four topics: (i) capacity building—providing opportunities on improving skills for scientists from RNAS^+^ member countries, (ii) funding support—sponsoring the activities of RNAS^+^, (iii) coordination—responsibility for the operation and development of RNAS^+^, and (iv) cooperation programs—initiating the application of multi-country joint collaborative projects in Asia. For each topic, several corresponding quantitative indicators were formed. In addition, to obtain the direct value of China’s contributions to this network, a score sheet on the above topics as well as for the overall contribution were created with scores ranging from “0” to “10.” The higher the score, the greater the contributions.

### Questionnaire and data collection

 An anonymous and web-based questionnaire on the above assessment research framework was designed as an assessment tool for evaluating China’s contributions to RNAS^+^. Three senior professionals of RNAS^+^ provided inputs to the content of the questionnaire and revised it, before the formal survey. This questionnaire had three parts: (i) general information of the participants; (ii) the core of the evaluation on China’s contributions to RNAS^+^ (Table 1), contained quantitative indicators and scoring tables on capacity building, funding support, coordination, cooperation program, and overall assessment, from the evaluation framework; and (iii) challenges and suggestions for RNAS^+^ development in the future. The questionnaire was used to collect the assessment information, and participants who frequently attended the recent RNAS^+^ annual workshops were invited to participate in this survey.

**Table 1:** Assessment framework of interview questions

Assessment framework	Assessment questions
Topic 1:Capacity building	1. How many times have the RNAS^+^ training courses been held in China?
	2. Have you ever attended the short-term training organized by China (except for the RNAS^+^ annual meeting/training)?
	3. Which skills did you learn from China (by workshops and trainings)?
Topic2: Funding	4. How many times have you received China’s grant to attend RNAS^+^ annual meeting/training?
	5. How many RNAS^+^ annual meetings / trainings were mainly sponsored by China but not held in China:
Topic 3: Coordination	6. Currently, there are already five committee chairmen of RNAS^+^. Do you know how many of them are Chinese?
	7. Have you ever been invited by China to be the instructor of RNAS^+^ training / the speaker of the annual meeting?
	8. Do you know the address of the RNAS^+^ website?
Topic 4: Cooperation program	9. Did you participate in the IDRC project?
	10. Are you willing to apply the new “Belt & Road Initiative” related program with Chinese experts?
Scoring sheet	Please indicate a score of 0 to 10 for China’s contributions / roles in RNAS^+^ (Description:"0”is no contribution, “10” is 100% contribution):
	11. China’s contributions / roles in improving capacity building of RNAS^+^?
	12. China’s funding contributions / roles to RNAS^+^?
	13. China’s contributions / roles in the coordination of RNAS^+^?
	14. China’s contributions / roles in applying for cooperation program / project jointly with RNAS^+^ member institutions?
	15. The overall assessment of China’s contribution / role in RNAS^+^?

RNAS^+^, Regional Network for Asian Schistosomiasis and Other Helminth Zoonoses; IDRC, International Development Research Centre

This web-based survey was distributed by e-mail to the participants who provided their e-mail address during the three recent RNAS^+^ annual workshops from 2017 to 2019. Each participant gave  a score for his/her understanding of RNAS^+^; the higher the value, the more familiar they were with RNAS^+^. In order to encourage the participants to complete the questionnaire, two reminders were sent to each participant who was also asked to send the link to their colleagues or friends who attended the RNAS^+^ annual workshops. The participants were asked to complete the questionnaire for once if they had attended more than one meeting.

### Data analysis

All original data of this web-based questionnaire were downloaded as a .sav file format from the back-stage management platform. Because all the questions in the web-based questionnaire were required, all the data were compliant with the integrity verification requirements. Data analysis was performed using IBM SPSS Statistics for Windows, version 26.0 (IBM Corp., Armonk, NY., US). Frequency and related percentage were reported to describe classified variables. The arithmetic average was reported for continuous variables, as well as a bar chart to present the numerical distributions. Highcharts were used to analyze the open-ended questions.

## Results

### General information

Of 71 participants who participated in the 2017, 2018, and 2019 RNAS^+^ annual meetings and provided e-mail addresses, 41 responded to the survey. The general information of the respondents is presented in Table 2. Twenty-seven participants were male; 85.37% (35/41) were aged > 35 years; most (37/41, 90.24%) were from the RNAS^+^ member countries; the remaining 4 (4/41, 9.76%) were international observers at RNAS^+^. The biggest number of respondents were from Indonesia (8/41, 19.51%), followed by China(7/41, 17.07%).

**Table 2:** Characteristics of the survey respondents

Variable	Description	Frequency	Percent(100%)
Gender	Male	27	65.85
	Female	14	34.15
	Total	41	100.00
Age (years)	≤35	6	14.60
	> 35	35	85.40
	Total	41	100.00
Education level	Diploma and below	2	4.88
	undergraduate	0	0.00
	Postgraduate	17	41.46
	Doctor	22	53.66
	Total	41	100.00
Nationality	Cambodia	5	12.20
	Indonesia	8	19.51
	Laos	1	2.44
	The Philippines	4	9.76
	Thailand	7	17.07
	Vietnam	3	7.32
	Myanmar	2	4.88
	China	7	17.07
	Others	4	9.76
	Total	41	100.00
Current employer	Government Authority	8	19.51
	University/ Research Institute	29	70.73
	Company / Enterprise	2	4.88
	Others: Retirement	2	4.88
	Total	41	100.00
Current focus area	Health management	1	2.44
	Diseases prevention and control	20	48.78
	Research and development technology	18	43.90
	Health policy	2	4.88
	Total	41	100.00

**Fig. 1:**
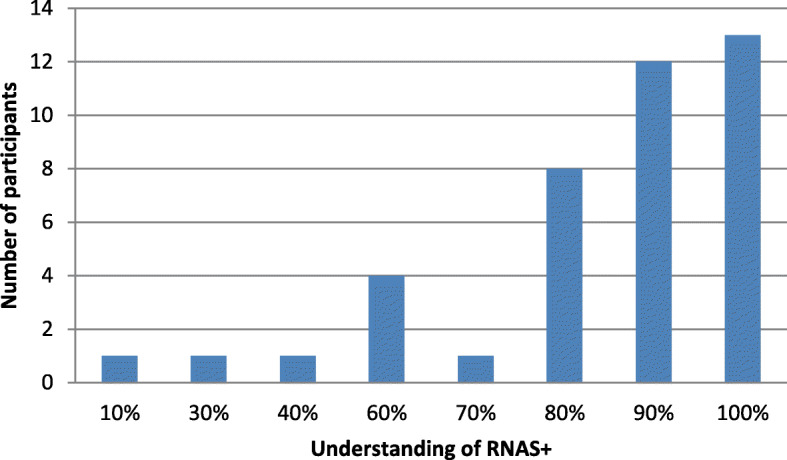
The participants understanding of RNAS^+^

As shown in Fig. 1, most of the respondents were familiar with RNAS^+^ (38/41,92.68%), their understanding of RNAS^+^ was greater than 60%, and only 3(3/41,7.32%) respondents had less than 40%. Importantly, 31.71% (13/41) indicated that they had 100% understanding of RNAS^+^, and the average value of understanding of RNAS^+^ was 83.17%.

### The overall assessment of China’s contributions to RNAS^+^

According to the survey, every participant gave a score to assess China’s contributions to RNAS^+^ (Table 3). The average score for the overall assessment of China’s contributions to RNAS^+^ was 8.81 (10 maximum score). The average score of China’s contributions to the improvement of capacity building, funding support, coordination, and the application of cooperation projects in RNAS^+^ were 8.92, 8.64, 8.75, and 8.67, respectively (10 for maximum score).

**Table 3:** Assessment score of China’s contributions to RNAS^+^ by survey respondents

Variable	Score*											Average score**
	0	1	2	3	4	5	6	7	8	9	10	
China’s contributions to improving of capacity building of RNAS^+^? N(%)^#^	0(0%)	0(0%)	0(0%)	0(0%)	0(0%)	0(0%)	0(0%)	4(11.11%)	8(22.22%)	11(30.56%)	13(36.11%)	8.92
China’s funding contributions to RNAS^+^? N(%)^#^	0(0%)	0(0%)	0(0%)	0(0%)	1(2.78%)	0(0%)	2(5.56%)	4(11.11%)	7(19.44%)	9(25%)	13(36.11%)	8.64
China’s contributions to the coordination of RNAS^+^? N(%)^#^	0(0%)	0(0%)	0(0%)	0(0%)	0(0%)	1(2.78%)	2(5.56%)	3(8.33%)	6(16.67%)	11(30.56%)	13(36.11%)	8.75
China’s contributions to applying for cooperation programme / project jointly with RNAS^+^ member institutions? N(%)^#^	0(0%)	0(0%)	0(0%)	0(0%)	0(0%)	2(5.56%)	1(2.78%)	3(8.33%)	7(19.44%)	11(30.56%)	12(33.33%)	8.67
The overall assessment of China’s contributions in RNAS^+^? N(%)^#^	0(0%)	0(0%)	0(0%)	0(0%)	0(0%)	0(0%)	2(5.56%)	2(5.56%)	8(22.22%)	13(36.11%)	11(30.56%)	8.81

* The scores given by the respondents about China’s contributions to RNAS^+^ from the questionnaire, “0 score” means no contribution, and “10 score” indicated 100% contribution. The higher the score, the more important China’s contributions to RNAS^+^

** Average score: the sum of all scores marked by the respondents / the total number of respondents

^#^ N(%): the number of respondents by each score (the number of respondents by each score/total number of respondents × 100%)

### China’s contributions in improving capacity building of RNAS^+^

**Fig. 2:**
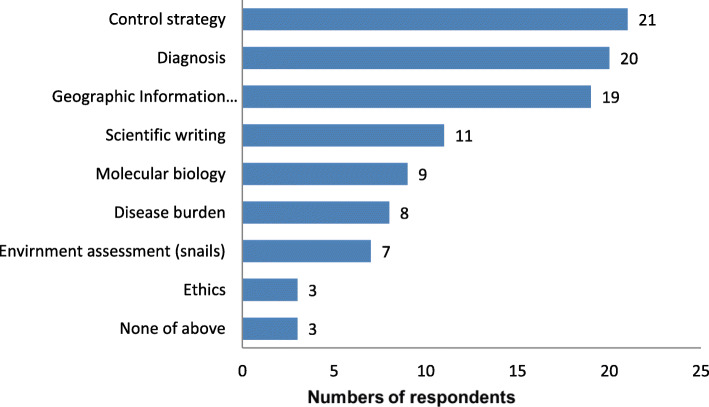
Skills learned from training activities held in China by survey respondents

According to the survey, four RNAS^+^ annual training courses were held in China. In addition, 7 (17.07%) respondents had attended the short-time training in China, including on snails control and schistosomiasis diagnosis skills. A total of 38 (92.68%) participants indicated that they had learned useful skills from training activities held in China, and the top three were control strategy, diagnosis, and geographic information system (GIS) (Fig. 2).

### China’s funding contributions to RNAS^+^

Most respondents (73.17%) had received China’s grants to attend RNAS^+^ annual workshops, and 26.83% (11/41) had received more than 5 times. In addition, China also sponsored the annual workshops held outside of China. Most respondents (70.73% ) attended the RNAS^+^ annual workshops mainly sponsored by China but held outside of China for more than once. Among respondents, 34.15% (14/41) said that China provided grants to support the annual workshops held outside of China at least 5 times.

### China’s contributions to the coordination of RNAS^+^

NIPD was one of the two institutions participating in the RNAS^+^ initiative. Chinese experts played important roles in the initiation and scale of the network. Up to now, two out of 5 experts had served as chairmen of RNAS^+^, 53.66% (22/41) of the respondents had invited/coordinated with China as instructors in the RNAS^+^ training courses or as the speaker at the annual workshops. China established the RNAS^+^ website and is responsible for website maintenance and information updates, while 68.29% (28/41) of the respondents know the address of the RNAS^+^ website.

### China’s contributions to the application of cooperation programs jointly running with RNAS^+^ member institutions

NIPD, along with national research institutes of the Philippines, Laos, Cambodia, and other countries, jointly applied and received two rounds of funds from the International Development Research Centre (IDRC), Canada, 36.59% (15/41) of the respondents attended this IDRC project. Most respondents, 82.93% (34/41) are willing to apply the new “Belt & Road Initiative”-related program with Chinese experts in the future.

### Challenges and suggestions for RNAS^+^ development

**Fig. 3:**
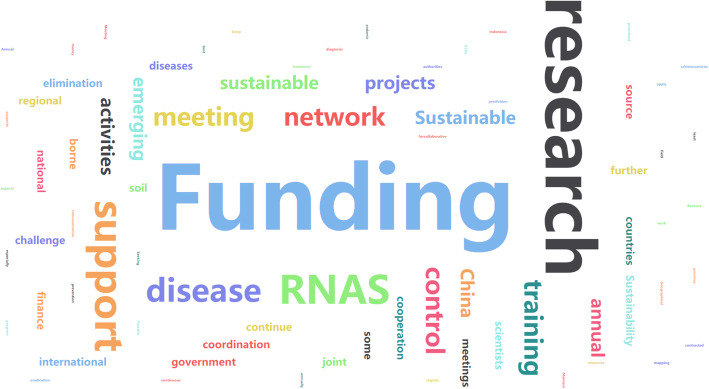
The highcharts of challenges confronting RNAS^+^ according to the survey participants

**Fig. 4:**
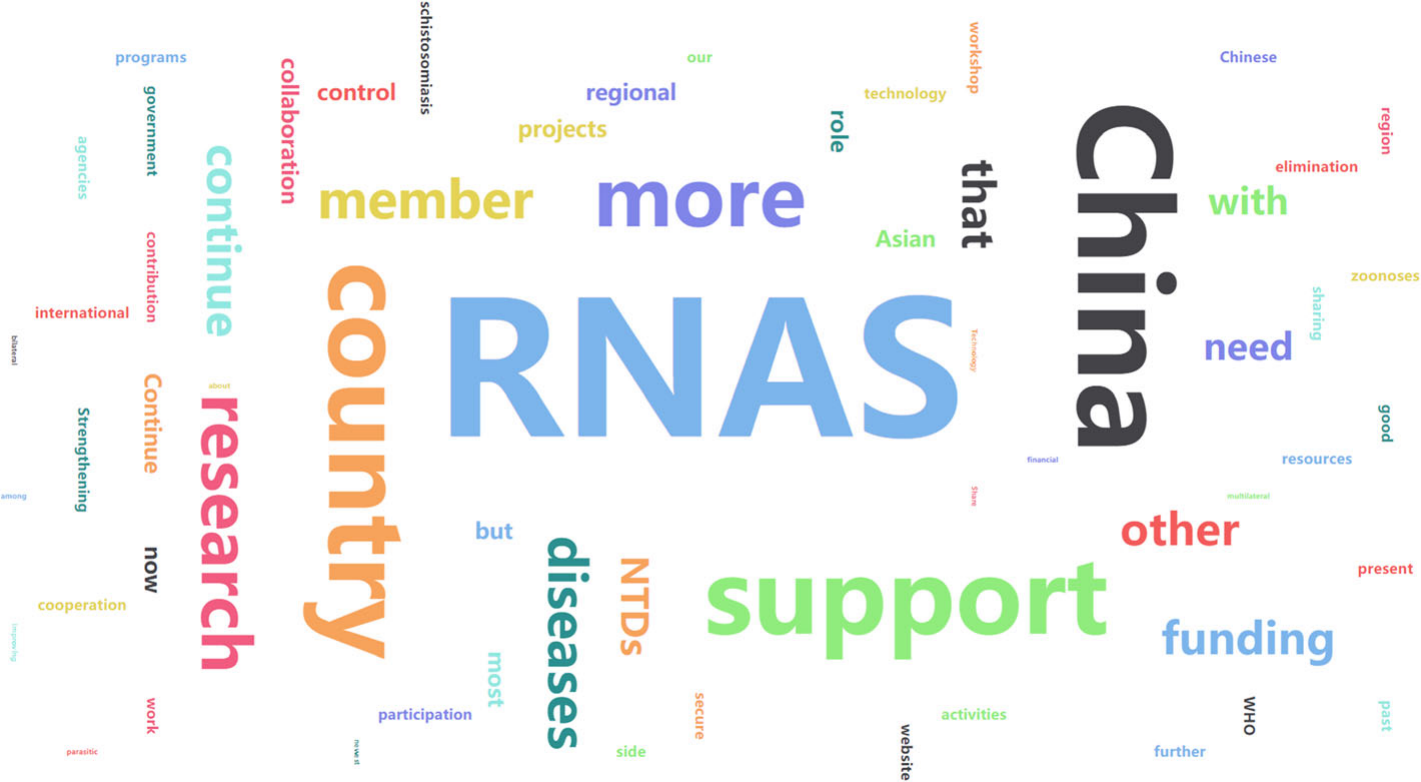
The highcharts of advice regarding China’s future role in RNAS^+^development according to the survey participants

From the survey, both the challenges faced by RNAS^+^ and some pieces of advice on what China should do in the future are summarized in Figs.3 and 4 by highcharts. First, the most important challenge of RNAS^+^ was lack of the sustainable financial support, and more than half of the participants (26/41) mentioned the word “funding” in the open-ended question. Second, some participants pointed out that there were some challenges with the research and diagnosis skills that need to be addressed on NTDs in the region. Other aspects, such as the emerging and re-emerging disease control as well as the multi-country collaborations among scientists and control authorities also need to be supported. Third, how to enhance the impact of this network and improve interregional conflicts are important issues that should be addressed.

Regarding China’s future contributions to RNAS^+^ development, most of the participants indicated that it is essential for China to continue her support in the following five areas: funding, research and technology, capacity building, and RNAS^+^ annual workshops as well as in training activities. In the future, the participants proposed that China should continually share in the work experience and resources, jointly apply bilateral/multilateral partnership projects with member countries, and expand more members and international agencies to RNAS^+^, to strengthen the operation of the network.

## Discussion

China, as one of two founding countries, has been actively engaged in the development of RNAS^+^. This paper offers a direct method to assess China’s contributions to the development of RNAS^+^. In this survey, through the scoring method, most participants gave a high score on China’s contributions to the development of RNAS^+^. This means that those participants agreed that China made great contributions and played an important role in the development of RNAS^+^. Combined with this survey and the literature review, Chinese contributions to the RNAS^+^ development can be concluded in several ways: (i) promoting fundraising, capacity building, and coordinating the affairs at the annual workshops and training courses organized by RNAS^+^ [4, 22]; (ii) sharing important experiences on China’s achievements on schistosomiasis and other parasitic diseases control [23, 24]; (iii) actively promoting the routine running of RNAS^+^ activities, including agenda setting and workshop reports [25]; (iv) directly participating in the training courses on instructions for schistosomiasis diagnosis, spatial informatics and others [4, 12]; (v) establishing and updating the RNAS^+^ website [26]; and (vi) playing the leading role in project cooperative studies, such as IDRC projects [27, 28], ultrasound diagnosis of *S. japonicum* infection [29], and epidemiological study on control strategy of schistosomiasis and liver fluke, and so on [24, 30].

A special issue was published to introduce the origin, progress, outcomes, challenges, and the way forward for the RNAS^+^ in the past 20 years [2, 4, 13–16, 31, 32]. RNAS^+^ has been operating as a platform for sharing information to enhance multi-country, multi-diseases, and multidisciplinary collaboration in the Asian region. As a result of the highly collaborative projects among member countries, RNAS^+^ has promoted the understanding of various aspects of schistosomiasis and other diseases, and emphasized that an integrated control approach can be established to eliminate the disease through intersectoral collaboration. On this issue, China’s contributions have also been affirmed on the four parts, such as capacity building [13], funding support [13], coordination [2, 15], and cooperation program [4, 14–16], of the development of RNAS^+^.

Additional challenges identified in the survey for the RNAS^+^ mainly focused on sustained funding, as well as research grants, which have also been reflected in recent RNAS^+^ publications [32]. RNAS^+^ has no sustainable funding by itself, nor fixed support from any large-scale transnational cooperation programs, indicating huge lack of financial support, and this  has been confirmed by most respondents who mentioned the necessity to increase financial support. Moreover, RNAS^+^ inspired research and cooperation projects are limited, and continuous skills and programs are needed to combat NTDs and emerging parasitic diseases in this region. Additionally, how to improve the impact of the network and how to deepen the cooperation are also the challenges. Undoubtedly, more donors are needed to assist the network in its current and future activities [31].

Several ways to enhance China’s contribution to RNAS^+^ development in the future were suggested. First, China has made great progress in schistosomiasis control in the past 70 years [33, 34], China should continually share its experiences to enhance cooperation within member countries as an important matter  listing in the profile agenda [35, 36]. Second, due to the need for capacity building by RNAS^+^ members, China can continue to support annual workshops and technical trainings as well as collaborative research activities [37, 38]. Third, China can motivate more institutions and international organizations to join and enlarge the institutional coverage of RNAS^+^. Fourth, the practice of RNAS^+^ suggests that establishing a network-type organization is a good approach, therefore, in addition to schistosomiasis and NTDs, other diseases requiring particular control with joint efforts in one specific region can also refer to this mode of networking for regional cooperation [39].

To our knowledge, this is the first attempt of an assessment on China’s contributions in regional networks, using an assessment questionnaire with the quantitative indicators in combination with four parts and scoring method. This is only one possible attempt to assess the contribution in the network. However, there are some limitations to this survey. Most of all, the number of participants is limited, and no responses were received from Japan and South Korea, which are two member countries of RNAS^+^, thus, the results may not represent those of all experts from all member countries. Moreover, this assessment was not carried out by an independent third party, which may have bias affected the results’ explanation to some extent. However, this survey has valuable reference on the role of China in the development of regional cooperation networking, e.g. RNAS^+^.

## Conclusion

This survey showed that China has played an important role in the development of RNAS^+^ network since its establishment in the fields of capacity building, funding support, coordination, and cooperation programs. China has effectively shared experience and provided professional support on technologies and resources to member countries during the past 20 years. The challenges of RNAS^+^ includes lack of sustainable funding, less research activities, few new skills promoted, and lower impacts at global level. China’s continual support is needed, especially in organizing annual workshops, capacity building, and cooperative research. The network-type organization, of RNAS^+^, which focuses on the cooperation for disease control and research, can yet be regarded as a great potential pattern for China to enhance regional cooperation. Thus, the research findings can be used to promote future cooperation between China and other RNAS^+^ member countries.

## Data Availability

Please contact author for data requests.

## Abbreviations

RNAS^+^: Regional Network for Asian Schistosomiasis and Other Helminth Zoonoses
RNAS: Regional Network on Asian Schistosomiasis
SDGs: Sustainable Development Goals
NTDs: Neglected Tropical Diseases
WHO: World Health Organization
NIPD: National Institute of Parasitic Diseases
China CDC: Chinese Center for Disease Control and Prevention
GIS: Geographic Information System
IDRC: International Development Research Centre
ASEAN: Association of Southeast Asian Nations
